# History-dependent phase transition character

**DOI:** 10.1140/epje/s10189-022-00221-2

**Published:** 2022-08-23

**Authors:** Juš Polanšek, Arbresha Holbl, Szymon Starzonek, Aleksandra Drozd-Rzoska, Sylwester J. Rzoska, Samo Kralj

**Affiliations:** 1 Faculty of Natural Sciences and Mathematics, University of Maribor, Koroska 160, 2000 Maribor, Slovenia; 2 Institute of High Pressure Physics Polish Academy of Sciences, ul. Sokołowska 29/37, 01-142 Warsaw, Poland; 3 Condensed Matter Physics Department, Jožef Stefan Institute, Jamova 39, 1000 Ljubljana, Slovenia

## Abstract

**Abstract:**

We consider history-dependent behavior in domain-type configurations in orientational order that are formed in configurations reached via continuous symmetry-breaking phase transitions. In equilibrium, these systems exhibit in absence of impurities a spatially homogeneous order. We focus on cases where domains are formed via (i) Kibble-Zurek mechanism in fast enough quenches or by (ii) Kibble mechanism in strongly supercooled phases. In both cases, domains could be arrested due to pinned topological defects that are formed at domain walls. In systems exhibiting polar or quadrupolar order, point and line defects (disclinations) dominate, respectively. In particular, the disclinations could form complex entangled structures and are more efficient in stabilizing domains. Domain patterns formed by fast quenches could be arrested by impurities imposing a strong enough random-field type disorder, as suggested by the Imry-Ma theorem. On the other hand, domains formed in supercooled systems could be also formed if large enough energy barriers arresting domains are established due to large enough systems’ stiffness. The resulting effective interactions in established domain-type patterns could be described by random matrices. The resulting eigenvectors reveal expected structural excitations formed in such structures. The most important role is commonly played by the random matrix largest eigenvector. Qualitatively different behavior is expected if this eigenvector exhibits a localized or extended character. In the former case, one expects a gradual, non-critical-type transition into a glass-type structure. However, in the latter case, a critical-like phase behavior could be observed.

**Graphical abstract:**

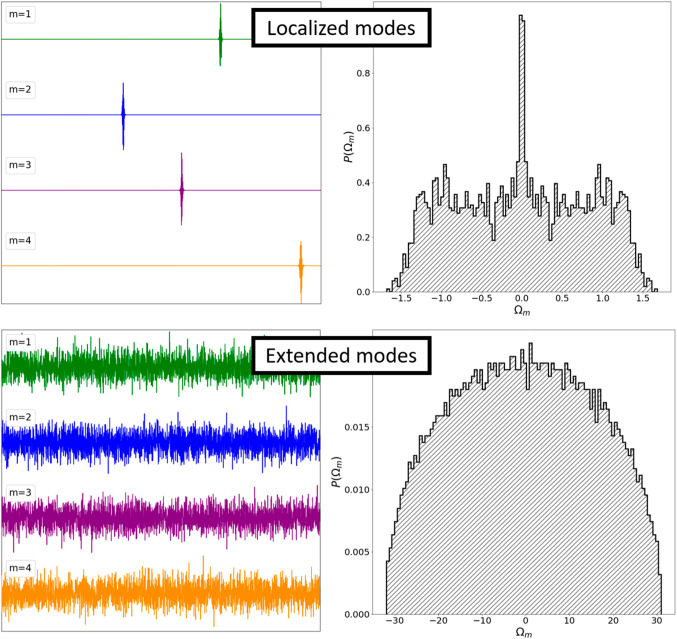

## Introduction

History-dependent configurations are ubiquitously formed in diverse physical systems. In particular, they might also appear in systems that in equilibrium display a relatively simple order in a phase that is entered via a symmetry-breaking phase transition [1]. Such systems often display kinetic-dependent qualitatively different configurations, where several features display universal behaviors which are independent of systems’ microscopic details [2, 3]. It is of strong interest to identify key parameters that dominate such behaviors.

Take for example discontinuous temperature-driven order–disorder phase transition, which in the lower symmetry phase exhibits (i) polar or (ii) quadrupolar (e.g., uniaxial nematic order) [4, 5]. The hallmark examples of these *p*-atic orientational order (where *p* = 1 and *p* = 2 refer to the polar and quadrupolar order, respectively) are (i) ferromagnetic–paramagnetic and (ii) isotropic-nematic liquid crystal (LC) phase transitions. The mesoscopic-scale order parameter could be in such cases represented by (i) vector $\overrightarrow{m}=m \widehat{s}$ and (ii) tensor $\underline{Q}=m(\widehat{s}\otimes \widehat{s}-\underline{I}/3)$ field [4], where $\left|\widehat{s}\right|=1$. Note that, these order parameters consist of two qualitatively different contributions: *amplitude m* and *phase* $\widehat{s}$. The amplitudes determine the strength of the established order, whereas phase fingerprints the symmetry-breaking choice. In the quadrupolar case, the states ±$\widehat{s}$ are physically equivalent. In bulk equilibrium, the symmetry broken phases are characterized by spatially homogeneous values of *m* and $\widehat{s}$. Furthermore, *m* exhibits a unique temperature-dependent value. On the other hand, $\widehat{s}$ displays infinite degeneracy [4] (i.e., any symmetry-breaking direction choice is equivalent).

However, complex configurations could be established on varying kinetic phase transition paths, particularly in the presence of *impurities* (i.e., different imperfections or the presence of “foreign” elements in the system). Let us first consider cases following phase transition quenches, where the phase change is realized in a relatively short time scale with respect to the relevant amplitude order parameter relaxation time [6]. In such cases, in well-enough separated regions, different symmetry-breaking directions are in general selected due to the finite speed of information propagation [6, 7]. Consequently, domains are formed, within which points along a similar direction. At domain interfaces, topological defects (TDs) are formed [8, 9], corresponding to topologically protected localized order parameter distortions. Due to their topological protection, they could be eliminated only via the annihilation [10, 11] of relevant defect-antidefect pairs if the field is not melted. Domain walls and TDs are in general energetically expensive. Consequently, in common cases, the characteristic linear size of domains grows with time [12, 13], which is enabled by the annihilation of TDs. Note that, qualitatively different TDs appear for polar- and quadrupolar-order parameter. The former can exhibit topologically stable point defects, while the latter can display in addition also line defects [4].

If the impact of impurities is relatively weak, the domain growth exhibits a universal scaling law ${\xi }_{d}\propto {t}^{\gamma }$, where $\gamma$ is the scaling coefficient [12–14]. For example, in three-dimensional (3D) nematics, it holds $\gamma \sim \frac{1}{2}$. But if the interaction with impurities is significant, they might arrest the domain size growth. Namely, phases reached via a symmetry-breaking phase transition possess energy costless Goldstone modes [4], which can be easily excited. According to the Imry-Ma theorem [15, 16], one of the pillars of statistical mechanics of disorder, even an infinitesimally weak random-field type of disorder destroys long-range order, which is anticipated in pure systems. The resulting structure is predicted to exhibit short-range order, where the characteristic Imry-Ma domain size ${\xi }_{d}^{(IM)}\propto {w}^{-2/(4-d)}$ is determined by the disorder strength *w* and *d* stands for the spatial dimensionality. Note that, subsequent investigations reveal that for weak enough disorder, quasi-long-range order or even short-range order might be established [17–20].

Finally, in slow enough quenches, where the system’s free energy barrier separating the competing phases at the phase transition temperature ${T}_{c}$ is large relative to ${{k}_{B}T}_{c}$ (${k}_{B}$ stands for the Boltzmann constant), supercooling of the higher symmetry phase could be realized. At low enough temperatures, the energy barrier (which is decreasing with *T*) could be overcome. Similarly, as for fast quenches, domain-type structures are expected to form. However, in supercooled cases, the relevant order parameter times are significantly shorter and the resulting ordering within domains is expected to be stiffer.

In summarizing, systems exhibiting a symmetry-breaking order–disorder phase transition are expected to exhibit domain-type configurations if they are reached via fast enough quenches or they are strongly supercooled in presence of impurities. If topological defects are strongly enough pinned [21] (i.e., the annihilation of TDs is hindered by energy barriers that are unlikely to be overcome by thermal fluctuations), domain-like structures could be stabilized for macroscopic scale time regimes, or even correspond to arrested metastable states protected by unsurmountable energy barriers. The effective interactions in such arrested configurations could be approximated by random-bond-like effective interactions. Furthermore, such systems are expected to exhibit several universal features, where some of which could be described by random matrices [22, 23]. In the following, we illustrate how such approaches could be implemented to analyze different configurational changes on varying relevant controlled parameters in systems of our interest.

The plan of the paper is as follows. In Sect. 2, we present common domain-type generic mechanisms. The resulting configurations experience randomness, and related effects are analyzed in Sect. 3. In Sect. 4, we present how these features could yield history-dependent phenomena. In the last section, we summarize the key features.

## Domain generating mechanisms

In the following, we illustrate two universal and qualitatively different domain generating mechanisms that are effective in configurations that are reached via a continuous symmetry-breaking transition. We present the Kibble [6], Kibble-Zurek mechanism [1], and the Imry-Ma theorem [15].

### Kibble and Kibble-Zurek mechanism

We first illustrate the derivation of the characteristic size ${\xi }_{d}^{(p)}$ of protodomains that are nucleated via the universal Kibble [6] and Kibble-Zurek (KZ) [1] mechanism. The Kibble mechanism was originally introduced in cosmology [6] in order to explain coarsening dynamics of topological defects in the Higgs field in the early universe. The principal aim of this and consequent latter investigations has been to study the impact of inflation velocity on the concentration of defects in the early universe because they might serve as nucleating sites for galaxies. The two conditions needed for the mechanism are (i) continuous symmetry-breaking and (ii) finite velocity of information propagation. Later, Zurek suggested [1] that critical slowing down should also play the important role. The resulting combined effects of (i) continuous symmetry-breaking, (ii) causality, and (iii) freezing of ordering close to the relevant phase transition are referred to as the Kibble-Zurek (KZ) mechanism. Validity and fundamental consequences of the KZ mechanism have been since 1990 intensively studied in various condensed matter systems [1, 7, 24–30], in which experiments can be realized and controlled. Namely, such experiments can not be realized on the cosmological scale.

Let us consider a fast enough temperature-driven order–disorder phase transition, where the symmetry broken phase exhibits a spatially homogeneous orientational order. For illustration, we consider the case where the established order is described by the vector order parameter $\overline{m} = m \hat{s}.$ Due to the finite speed of information in well-separated regions, the symmetry-breaking directions are not correlated. Consequently, a domain-type pattern is formed. Here, a domain refers to a region characterized by a similar ordering (i.e., orientations of $\widehat{s}$) within it. At domain intersection, there is large probability that TDs will be formed. To a good approximation, the established pattern can be described by a single characteristic length ${\xi }_{d}$. Note that, the average separation between nearby defects is also given by ${\xi }_{d}$. This is the essence of the Kibble mechanism. The first formed domains are referred to as the *protodomains*. Derivation of their characteristic size is described by the KZ mechanism.

Originally, the KZ mechanism was derived for the temperature-driven second-order phase transition [1, 6]. The temperature variations are described by the dimensionless temperature

1 $$ r = {{\left( {T - T_{c} } \right)} \mathord{\left/ {\vphantom {{\left( {T - T_{c} } \right)} {T_{c} }}} \right. \kern-\nulldelimiterspace} {T_{c} }}, $$

where ${T}_{c}$ stands for the phase transition temperature. Let us assume linear time variation in temperature across ${T}_{c},$ which is characterized by the quench rate ${\tau }_{Q}:$

2 $$ t = - \tau_{Q} r. $$

where ${\tau }_{Q}$ describes the time needed to increase the temperature from *T* = 0 to ${T}_{c}$. Close to the second-order phase transition, the characteristic amplitude-order parameter relaxation length and relaxation time obey equations

3 $$ \tau \approx \frac{{\tau_{0} }}{{\left| r \right|^{\eta } }} ,\quad \xi \approx \frac{{\xi_{0} }}{{\left| r \right|^{v} }}. $$

where $\eta$ and $v$ stand for the universal critical coefficients, and ${\tau }_{0}$ and ${\xi }_{0}$ determine characteristic responses deep in the symmetry broken phase. For example, the mean-field description of the paramagnetic–ferromagnetic second-order transition yields [1, 4] $\eta =1$ and $v=1/2.$

To estimate the size of *protodomains,* we assume that we start from the disordered phase, corresponding to $$ r>0$$ (see Eq. 1). Then we linearly in time approach the phase transition according to Eq. (2). The maximal size of fluctuations exhibiting local order within the disordered “sea” is estimated by $$\xi $$ (see Eq. 3). The regime corresponding to $$\left|t\right|>\tau $$ is referred to as the “impulse” regime, where the dynamic of the system is fast enough to adapt to changes in temperature. Therefore, in this regime, we assume that the system exhibits roughly equilibrium ordering. The qualitative change in behavior is estimated by the condition, when the time to reach the phase transition, referred to as the Zurek time $${t}_{z},$$ becomes comparable to the relaxation time. Thus, the Zurek time is defined by the condition [1]

4 $$ \left| {t_{z} } \right| = \tau , $$

Taking into account Eqs. (2) and (3), it follows

5 $$ \left| {t_{z} } \right|\sim \left( {\tau_{0} \tau_{Q}^{\eta } } \right)^{{\frac{1}{1 - \eta }}} . $$

In the time regime $- \left| {t_{z} } \right| < t < \left| {t_{z} } \right|$, to which we referred as the adiabatic regime, the order parameter dynamics is relatively slow. In our approximate treatment, we set that dynamics is frozen in in this time interval and that the system falls out of equilibrium. When the system exits the adiabatic regime at $t = \left| {t_{z} } \right|$, the dynamics unfreezes. Note that, at $t = - \left| {t_{z} } \right|$, the size of the largest fluctuation generated clusters exhibiting ordering is estimated by

6 $$ \xi^{{\left( {\max } \right)}} = \xi \left( {\left| {r_{z} } \right| = \frac{{\left| {t_{z} } \right|}}{{\tau_{Q} }}} \right). $$

For temperatures, corresponding to $$ t< 0$$, such clusters are unfavorable. One assumes that on crossing the temperature interval {−$$\left|{r}_{z}\right|$$,$$\left|{r}_{z}\right|$$}, corresponding to the adiabatic regime, the correlation length is frozen due to dynamical slowing down. On exiting this interval, the fluctuations unfreeze at $$=\left|{t}_{z}\right|$$. At the corresponding temperature, the clusters exhibiting orientational order become energetically favorable and tend to expand. Therefore, the initial size $${\xi }_{p}$$ of domains, commonly referred to as the *protodomains*, is estimated by $${\xi }_{p}={\xi }^{(\mathrm{max})}$$. It follows [1]

7 $$ \xi_{p} \sim \xi_{0} \left( {\frac{{\tau_{Q} }}{{\tau_{0} }}} \right)^{{\frac{v}{1 + \eta }}} . $$

For $\eta = 1$ and $v=1/2,$ one obtains [30]

8 $$ \left| {t_{z} } \right|\sim \sqrt {\tau_{0} \tau_{Q} } ,\,\xi_{p} = \xi_{0} \left( {\frac{{\tau_{Q} }}{{\tau_{0} }}} \right)^{1/4} . $$

### Imry-Ma theorem

We next consider a possible static source of disorder. The Imry-Ma theorem [15] claims that even an infinitesimally weak random-field type disorder breaks the system into a domain-type pattern exhibiting short-range order. The corresponding characteristic domain length ${\xi }_{d}^{(\mathrm{IM})}$ reveals the compromise between elastic and random-field tendencies. The former favors spatially homogeneous order and the latter tends to align the nematic field along a local random-field enforced orientation.

To express ${\xi }_{d}^{(\mathrm{IM})}$ in terms of material parameters, let us consider an average domain of volume *V*_d_ and estimate the key free energy contributions within it. We use a minimal continuum type model describing an ensemble of spins $\widehat{s}$, where $\left|\widehat{s}\right|=1.$ We set that they tend to be aligned homogeneously along a symmetry-breaking direction in absence of disorder. In addition, we introduce a random-field term that enforces locally randomly selected orientation $\widehat{e}$, and $\left|\widehat{e}\right|=$ The resulting free energy density is expressed [15, 20] as $f = f_{e} + f_{{{\text{RF}}}} :$

9a $$ f_{e} = \frac{K}{2}\left| {\nabla \hat{s}} \right|^{2} , $$

9b $$ f_{{{\text{RF}}}} = - \frac{W}{2}P_{n} \left( {\hat{e} \cdot \hat{s}} \right). $$

The elastic term ${f}_{e}$ enforces homogeneous orientational order and is weighted by a positive elastic constant $K.$ The random-field contribution ${f}_{RF}$ is weighted by a positive constant $W$, ${P}_{n}$ stands for the Legendre polynomial of order n and enforces locally orientation along $\widehat{e}.$ The cases *n* = 1 (${P}_{1}\left(x\right)=x$) and *n* = 2 (${P}_{2}\left(x\right)=\left(3{x}^{2}-1\right)/2$) refer to systems exhibiting polar and nematic orientational order, respectively.

We focus on an average domain of volume ${V}_{d}\sim {\left({\xi }_{d}\right)}^{d}$, where ${\xi }_{d}$ stands for the characteristic linear domain size. The corresponding average domain free energy penalty is approximated by [20]

10 $$ \Delta F_{d} \sim \frac{{V_{d} }}{2}\left( {\frac{K}{{\xi_{d}^{2} }} - W\overline{{P_{n} \left( {\hat{e} \cdot \hat{s}} \right)}} } \right). $$

where the overbar $\overline{(\dots )}$ stands for the spatial average within ${V}_{d},$ whose average orientation is determined by$\widehat{s}$. According to the central limit theorem, it holds [4, 15] $\overline{{P_{n} \left( {\hat{e} \cdot \hat{s}} \right)}} \sim {1 \mathord{\left/ {\vphantom {1 {\sqrt {N_{d} } }}} \right. \kern-\nulldelimiterspace} {\sqrt {N_{d} } }}$, where ${N}_{d}\sim {\left({\xi }_{d}/{a}_{\mathrm{RF}}\right)}^{d}$ estimated number of $\widehat{e}$ random reorientations within ${V}_{d},$ where the average separation of nearby reorienting sites is given by ${a}_{RF}.$ The size ${\xi }_{d}^{(\mathrm{IM})}$ is obtained by balancing the elastic and random-field interactions. From the requirement $\Delta {F}_{d}=0$, one obtains [15, 20]

11 $$ \xi_{d}^{{\left( {{\text{IM}}} \right)}} \sim \left( {\frac{K}{{Wa_{{{\text{RF}}}}^{d/2} }}} \right)^{{\frac{2}{4 - d}}} . $$

Therefore, in 2D and 3D systems, it holds ${\xi }_{d}^{(\mathrm{IM})}\sim \frac{K}{W{a}_{\mathrm{RF}}}$ and ${\xi }_{d}^{(\mathrm{IM})}\sim {\left(\frac{K}{W{a}_{\mathrm{RF}}^{3/2}}\right)}^{2}$, respectively.

## Random-field favored configurations

We further discuss nature of excitations [4, 22, 23, 31] that are enabled by effective interactions within the system. For illustrating purposes, we use the simplest possible toy model.

We originate from the Ising lattice model in a transverse field in the Cartesian system (*x*, *y*, *z*), which is defined by the unit vector triad ($\hat{e}_{x} ,\hat{e}_{y} ,\hat{e}_{z}$). The local orientation at the *i*-th site is determined with pseudospins ${\widehat{s}}_{i},$ to which we henceforth refer to as *spins*. Furthermore, in domain-type patterns, these *spins* represent average orientation within each domain. Complex interdomain interactions are described by interaction matrix$\underline{J}$. The matrix component ${J}_{ij}$ describes the interaction between *i*-th and *j*-th *spin* (*i.e.*, domain). We express the dimensionless interaction energy of *N*-interacting spins as [32–34]

12 $$ W = - \frac{1}{2}\sum\limits_{i} {\sum\limits_{j} {J_{ij} s_{i}^{z} s_{j}^{z} - \Omega \sum\limits_{i} {s_{i}^{x} .} } } $$

We treat ${\widehat{s}}_{i}$ as a classical unit vector and we use XY-type model, where the orientation of *spins* is confined to the Cartesian (*x*, *z*) plane. Cases ${J}_{ij}>0$ (${J}_{ij}<0$) favor parallel (antiparallel) alignment of *spins* along the *z*-axis. The transverse field $\Omega$ plays the role of an external ordering field $\overrightarrow{\Omega }=\Omega {\widehat{e}}_{x}.$ In the limit $\Omega \to \infty$ and finite values of ${J}_{ij}$, spins are homogeneously aligned along ${\widehat{e}}_{x}.$

Furthermore, in our approximate treatment, we analyze conditions at zero temperature (i.e., we neglect thermal fluctuations). We parametrize *spins* in terms of angles ${\theta }_{i}$ (*i.e.*, scalars ${u}_{i}=cos{\theta }_{i}$) as

13 $$ \hat{s}_{i} = \cos \theta_{i} \hat{e}_{x} + \sin \theta_{i} \hat{e}_{z} = u_{i} \hat{e}_{x} + \sqrt {1 - u_{i}^{2} } \hat{e}_{z} . $$

In the following, we study how ordering imposed by interactions ${J}_{ij}$ is established on decreasing $\Omega$ where we start from the limit $\Omega \to \infty$, where it holds ${\widehat{s}}_{i}={\widehat{e}}_{x}$(i.e., ${u}_{i}=0$). We focus on the regime where the *spins* begin to depart from the *x*-axis alignment, and we assume $\left|{u}_{i}\right|\ll 1.$ Consequently, we can expand *W* in terms of small values of ${u}_{i}.$

It follows

14 $$ W\sim - \frac{1}{2}\sum\limits_{i} {\sum\limits_{j} {J_{ij} u_{i} u_{j} } } - {\Omega }\sum\limits_{i} {\left( {1 - \frac{{u_{i}^{2} }}{2} - \frac{{u_{i}^{4} }}{8}} \right)} = W^{\left( 2 \right)} + \frac{{\Omega }}{8}\sum\limits_{i} {u_{i}^{4} ,} $$

where we took into account all the terms up to the fourth order in ${u}_{i}.$

Note that, the minimization of ${W}^{\left(2\right)}$ (i.e., harmonic term) yields the equilibrium equation $\sum_{j}{J}_{ij}{u}_{j}=\Omega {u}_{j},$ which we express in the matrix notation as

15 $$ \underline {J} \hat{u}^{\left( N \right)} = \Omega \hat{u}^{\left( N \right)} . $$

where the *N*-dimensional vector ${\widehat{u}}^{(N)}=({u}_{1},{u}_{2},{u}_{3},\dots {u}_{N})$ approximates the configuration of spins that are slightly misaligned from the ${\widehat{e}}_{x}$ direction, and $\underline{J}=\{{J}_{ij}\}$ stands for the interaction matrix. Equation (5) is solved for eigenvectors ${\widehat{u}}^{(m)}$ of $\underline{J}$:

16 $$ \underline {J} \hat{u}^{\left( m \right)} = {\Omega }_{m} \hat{u}^{\left( m \right)} . $$

where ${\Omega }_{m}$ stands for eigenvalues, and eigenvectors are normalized and orthogonal. It holds

17 $$ \sum\limits_{i} {u_{i}^{\left( m \right)} u_{i}^{\left( n \right)} = \delta_{mn} , } $$

and ${\delta }_{mn}$ stands for the Kronecker symbol.

For relatively large values of $\Omega ,$ it is sensible to expand ${\widehat{u}}^{(N)}$ in terms of $\underline{J}$ eigenvectors as

18 $$ \hat{u}^{\left( N \right)} = \sum\limits_{m} {A_{m} \hat{u}^{\left( m \right)} } , $$

where ${A}_{m}$ is the measure amplitudes of modes.

Taking into account the expansion Eq. (18) and ortho-normal condition Eq. (17) in the expression for *W* (Eq. (14)), we get a Landau-type expansion in terms of excitation mode amplitudes

19 $$ W = - N\Omega + \frac{1}{2}\sum\limits_{m} {A_{m}^{2} \left( {\Omega - \Omega_{m} } \right)} + \frac{1}{4}\sum\limits_{m} {\sum\limits_{n} {\sum\limits_{k} {\sum\limits_{l} {\sigma_{mnkl} A_{m} A_{n} A_{k} A_{l} } } } } , $$

where

20 $$ \sigma_{mnkl} = \frac{{\Omega }}{2}\sum\limits_{i} {u_{i}^{\left( m \right)} u_{i}^{\left( n \right)} u_{i}^{\left( k \right)} u_{i}^{\left( l \right)} } . $$

### Configurational changes on varying ${\varvec{\Omega}}$

In the following, we study configurational evolution on decreasing the external ordering field strength $\Omega .$ In the first step, we neglect the interaction among competing modes. Afterward, we analyze the interaction between two modes and estimate threshold conditions leading to structural transformations.

#### Independent modes

We first neglect couplings among modes. It follows

21 $$ W\sim - N{\Omega } + \sum\limits_{m} {\left( {\frac{1}{2}A_{m}^{2} \left( {{\Omega } - {\Omega }_{m} } \right) + \frac{1}{4}A_{m}^{4} \sigma_{m} } \right)} , $$

where we introduced quantities

22 $$ \sigma_{m} = \frac{{\Omega }}{2}F_{4}^{\left( m \right)} , $$

23 $$ F_{n}^{\left( m \right)} = \sum\limits_{i} {u_{i}^{\left( m \right)n} ,} $$

where $n\in \{\mathrm{1,2},4\}$. Note that ${\sigma }_{m}>0$ and ${F}_{2}^{(m)}=1$ because to modes are normalized.

Minimization of *W* with respect to mode amplitudes yields

24 $$ A_{m}^{\left( 0 \right)} \left[ {\Omega > \Omega_{m} } \right] = 0, \,A_{m}^{\left( 0 \right)} \left[ {\Omega \le \Omega_{m} } \right] = \sqrt {\left( {\Omega_{m} - \Omega } \right)/\sigma_{m} } . $$

where the superscript (0) indicates that the modes are decoupled. It follows that on decreasing $\Omega$, the *m*-th mode condensates via a second-order-type structural transformation at $\Omega ={\Omega }_{m}$. The total response of the system along the *z*-axis is given by Eq. (18).

#### Coupled modes

Next, we consider coupling among the modes, which is in general enabled by the last (fourth-order) term of Eq. (14). For analytical purposes, we limit to cases when only two modes mutually compete, i.e.:

25 $$ \begin{aligned} W = & - N\Omega + \frac{1}{2}\sum\limits_{m} {A_{m}^{2} \left( {\Omega - \Omega_{m} } \right)} \\ & + \frac{1}{4}\sum\limits_{m} {\left( {\sigma_{m} A_{m}^{4} + \mathop \sum\limits_{l \ne m} \left( {4\mu_{ml} A_{m} A_{l}^{3} + 3\gamma_{ml} A_{m}^{2} A_{l}^{2} } \right)} \right)} , \\ \end{aligned} $$

26 $$ \mu_{ml} = \frac{\Omega }{2}\sum\limits_{i} {u_{i}^{\left( m \right)} u_{i}^{\left( l \right)3} } ,\,\gamma_{ml} = \frac{\Omega }{2}\mathop \sum \limits_{i} u_{i}^{\left( m \right)2} u_{i}^{\left( l \right)2} . $$

For $\Omega >0$, it holds ${\sigma }_{m}>0$ and ${\gamma }_{ml}>0.$ On the contrary, contributions ${\mu }_{ml}$ might be also negative. Furthermore, these terms are in general smaller with respect to ${\sigma }_{m}$ and ${\gamma }_{ml}$ values because the summing contributions in ${\mu }_{ml}$ could be of opposite sign. Despite this, the latter terms might affect the qualitative behavior of the system and give rise to hysteresis behavior. Namely, these terms are the only contributions in Eq. (25) that are sensitive to relative orientation (i.e., the sign of amplitudes) of modes.

An equilibrium (meta) stable configuration, in which two (i.e., *m*-th and *l*-th) modes are opened, satisfies equations $\frac{\partial W}{\partial {A}_{m}}=\frac{\partial W}{\partial {A}_{l}}=0$, $\frac{{\partial }^{2}W}{\partial {A}_{m}^{2}}=\frac{{\partial }^{2}W}{\partial {A}_{l}^{2}}>0$, and $\frac{{\partial }^{2}W}{\partial {A}_{m}^{2}}\frac{{\partial }^{2}W}{\partial {A}_{l}^{2}}-{\left(\frac{{\partial }^{2}W}{\partial {A}_{m}\partial {A}_{l}}\right)}^{2}>0.$ The condition, where such a configuration becomes unstable, is determined by

27 $$ \left( {\frac{{\partial^{2} W}}{{\partial A_{m} \partial A_{l} }}} \right)^{2} - \frac{{\partial^{2} W}}{{\partial A_{m}^{2} }}\frac{{\partial^{2} W}}{{\partial A_{l}^{2} }} = 0 . $$

where

28a $$ \frac{\partial W}{{\partial A_{m} }} = \left( {\Omega - \Omega_{m} } \right)A_{m} + \sigma_{m} A_{m}^{3} + \mu \left( {A_{l}^{3} + 3A_{l} A_{m}^{2} } \right) + 3\gamma A_{m} A_{l}^{2} , $$

28b $$ \frac{{\partial^{2} W}}{{\partial A_{m}^{2} }} = \left( {{\Omega } - {\Omega }_{m} } \right) + 3\sigma_{m} A_{m}^{2} + 6\mu A_{m} A_{l} + 3\gamma A_{l}^{2} , $$

28c $$ \frac{{\partial^{2} W}}{{\partial A_{m} \partial A_{l} }} = 3\mu \left( {A_{l}^{2} + A_{m}^{2} } \right) + 6\gamma A_{m} A_{l} , $$

and for simplicity, we set $\gamma \sim \gamma_{ml}$, and $\mu \sim \mu_{ml} .$

#### Destabilization of parallel and “antiferromagnetically” coupled localized modes

Next, we derive an estimate for the critical condition for which “antiferromagnetic” coupled (i.e., $\mu >0$) modes become unstable. For illustration purposes, we set that the mode amplitudes are equal, i.e., ${A}_{l}={A}_{m}=A.$ We originate from the cubic equation $\frac{\partial W}{\partial {A}_{m}}=0$ (see Eq. 28a) and we solve it by expanding $A$ about the independent mode solution (see Eq. 24). It follows

29 $$ A\sim A^{\left( 0 \right)} \left( {1 - \frac{4\mu + 3\gamma }{{2\beta }}} \right), $$

where $\beta ={\beta }_{m}\sim {\beta }_{l}.$ This equation suggests that the coupling between the modes diminishes their amplitude. We insert this results into the stability condition Eq. (27), which yields the critical condition

30 $$ \beta = 6\left( {\mu + \gamma } \right). $$

This condition could be fulfilled only if the overlap between the modes is large enough. To illustrate this, we set that both modes are localized and we model them using a one-dimensional ansatz

31 $$ u_{i}^{\left( m \right)} \sim c_{m} e^{{ - \left| {x_{i} - x_{m} } \right|/\xi_{m} }} . $$

where ${x}_{i}$ determines the *i*-th spin site, which is localized at ${x}_{m},$ ${\xi }_{m}$ describes its localization length, and ${c}_{m}$ in an amplitude (note that, the normalization condition Eq. (17) should be fulfilled). In our estimate, we set that the modes have the same shape, i.e., $\xi \equiv \xi_{m} \sim \xi_{l} .$ Using the ansatz Eq. (31) in Eq. (30) yields the condition

32 $$ \left| {x_{i} - x_{m} } \right|/\xi \sim 0.7. $$

This relation reveals under what circumstances an initially parallel (i.e., they both have positive amplitudes) modes which are “antiferromagnetically” coupled become unstable. One sees that the instability occurs if they are close enough with respect to their localization length.

#### Mutual immobilization of coupled extended modes

We next analyze coupling between extended (i.e., nonlocalized) modes. We show that on decreasing $\Omega ,$ in general, the first extended opened mode hinders opening of the later extended modes.

To illustrate this, we consider value of $\Omega$ where only one mode (${A}_{m}={A}_{1}$) is open, and conditions are such that it is extended. To estimate its amplitude, we assume that its structural configuration is Gaussian. It follows ${\mathrm{F}}_{4}^{(1)}\sim \frac{3}{N}$ and (see Eq. 24)

33 $$ A_{1}^{\left( 0 \right)} \sim \sqrt {\frac{{2N\left( {\Omega_{1} - \Omega } \right)}}{3}} . $$

Then, we decrease $$\Omega $$ slightly below the second largest eigenvalue $${\Omega }_{2}.$$ In this case, the system energy is determined by Eq. (25), where only two modes are open (i.e., $${A}_{m}={A}_{1}, {A}_{l}={A}_{2})$$, and we neglect the term weighted by $${\mu }_{ml}.$$ In the equilibrium, it holds

34 $$ 0 = \frac{\partial W}{{\partial A_{2} }}\sim \left( {\Omega - \Omega_{2} } \right)A_{2} + \beta A_{2}^{3} + 3\gamma A_{1}^{2} A_{2} , $$

where we assume $\beta = \beta_{1} \sim \beta_{2}$ and $\gamma = \gamma_{1} \sim \gamma_{2} .$ Nonzero solutions of Eq. (23) read

35 $$ A_{2} = \sqrt {{{\left( {{\Omega }_{2} - {\Omega } - 3{\gamma A}_{1}^{2} } \right)} \mathord{\left/ {\vphantom {{\left( {{\Omega }_{2} - {\Omega } - 3{\gamma A}_{1}^{2} } \right)} \beta }} \right. \kern-\nulldelimiterspace} \beta }} . $$

Let us set $\Omega \sim {\Omega }_{2}$ and for Gaussian distribution of mode components, one obtains $\gamma \sim\Omega /(2\mathrm{N})$ and it follows

36 $$ A_{2}^{2} \sim \frac{2N}{{3\Omega }}\left( {\Omega_{2} - \Omega_{1} } \right) < 0. $$

Thus, the second mode cannot open.

### Eigenvector and eigenvalues of the interaction matrix

Below, we illustrate numerically behavior which is commonly observed in systems whose key features are described by random matrices [34–36]. We claim that the latter well describe dominant properties in domain-type configurations.

For sake of simplicity, we limit to one-dimensional systems, where we consider different ranges of interactions. In such a way, we mimic roughly higher-dimensional systems in which systems’ elements interact with a larger number of neighbors. As testbed systems, we treat cases where (i) first neighbors, (ii) *n*-neighbors, and (iii) all *N* elements (*i.e.*, infinite range) interact. We remind that in domain-type patterns, *spins* represent “scaled” objects (*i.e.*, the *i*-th *spin* ${\widehat{s}}_{i}$ fingerprints the average orientation of the *i*-th domain). Note that, the stability of domains results from pinned topological defects [8, 37, 38]. Different types of localized topologically stable defects that can be present are determined by the symmetry of the system [4, 39, 40]. For example, systems described by a vector orientational-order parameter (e.g., magnetic or electric ordering) can exhibit only point defects. On the other hand, systems exhibiting nematic symmetry possess also line defects, which present in general domain-type patterns most abundant [12] TDs. In cases dominated by point defects, one expects relatively strong coupling only among adjacent domains. On the other hand, in presence of line defect, also, relatively well-separated domains could be correlated, giving rise to an effectively longer range of order.

As a reference, we consider an unperturbed system in which the nonzero pair interactions are equal to ${J}_{ij}=J\equiv 1.$ In these cases in the equilibrium, all the *spins* (independent of the range of interaction) are aligned along the positive or negative *z*-axis direction. This solution equals the $\underline{J}$ eigenvector corresponding to the largest eigenvalue. In addition, we consider also random matrices. We study two qualitatively different random distributions in ${J}_{ij}$ values. We either randomly chose ${J}_{ij}$ values in the interval [0, 1] or [− 1, 1]. We refer to these cases as “glass” and “spin-glass” matrices, respectively.

The structural details of the mode eigenvectors can be inferred from values of ${F}_{1}^{(m)}$ and ${F}_{4}^{(m)}$. Let us assume that ${N}^{(m)}$ spins are “opened” (i.e., the orientation of these *spins* deviates from the *x*-axis) in the *m*-th mode. We henceforth refer to such an assembly as a *cluster* if ${N}^{(m)}\ll N$ and the opened sites are connected. Owing to the normalization of modes, it roughly holds ${u}_{i}^{(m)}\sim \pm 1/\sqrt{{N}^{(m)}}$ (i.e., ${F}_{2}^{(m)}=1$). Consequently, the value of ${F}_{4}^{(m)}$ fingerprints the *m*-th mode *cluster* size:

37 $$ 1/F_{4}^{\left( m \right)} \sim N^{\left( m \right)} . $$

Furthermore, the value of ${F}_{1}^{(m)}$ is expected to be relatively small with respect to $\sqrt{{N}^{(m)}}$ if the mode exhibits oscillations about zero.

**Fig. 1:**
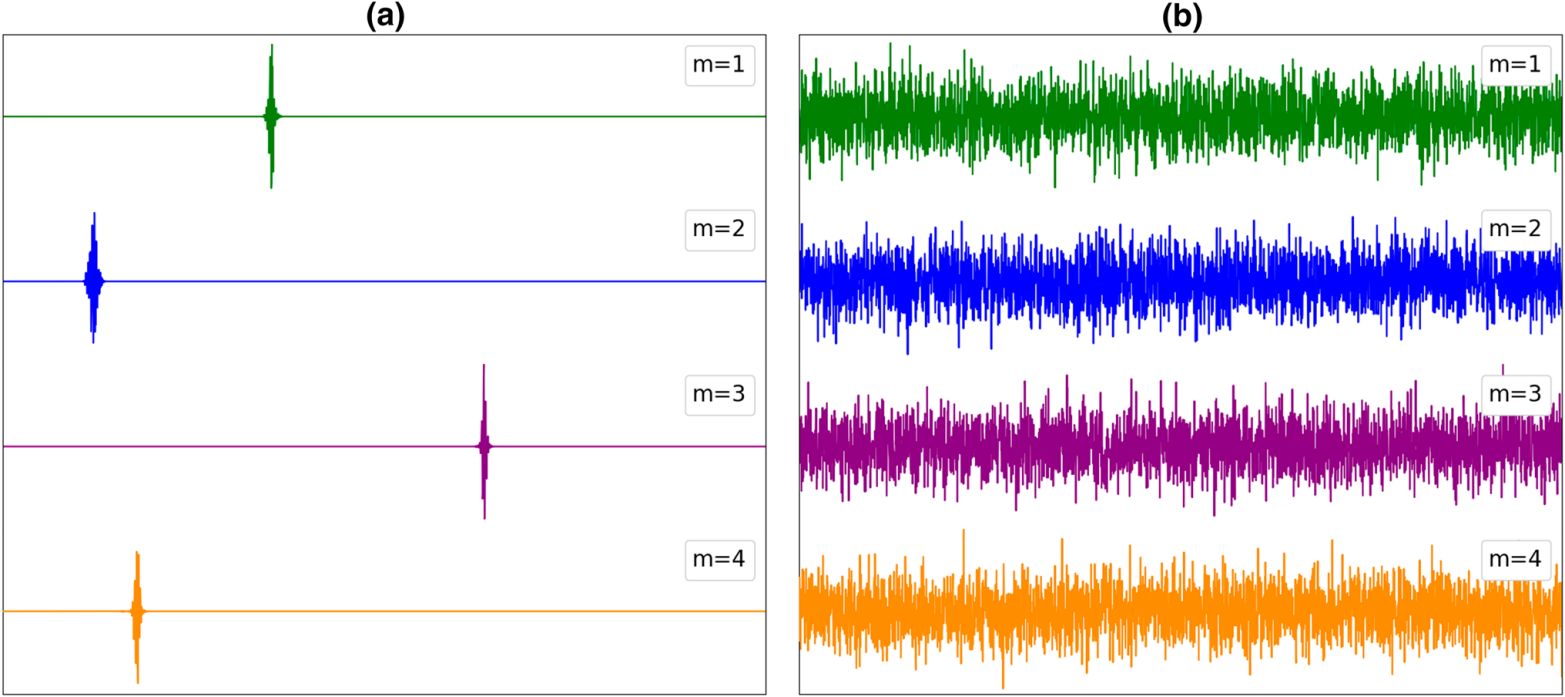
First four opened modes ${\widehat{u}}^{(m)}$ (corresponding to the first, second, third, and fourth largest eigenvalue) for **a** four first neighbors, and **b** N coupled neighbors (infinite interaction range). ${J}_{ij}\in \left[\mathrm{0,1}\right],$ 1D, *N* = 3000. The plot shows values of ${u}_{i}^{(m)}$ along the *x*-axis. The vertical axis is scaled in arbitrary units (i.e., $\sum_{i}{u}_{i}^{(m)2}=1)$

**Fig. 2:**
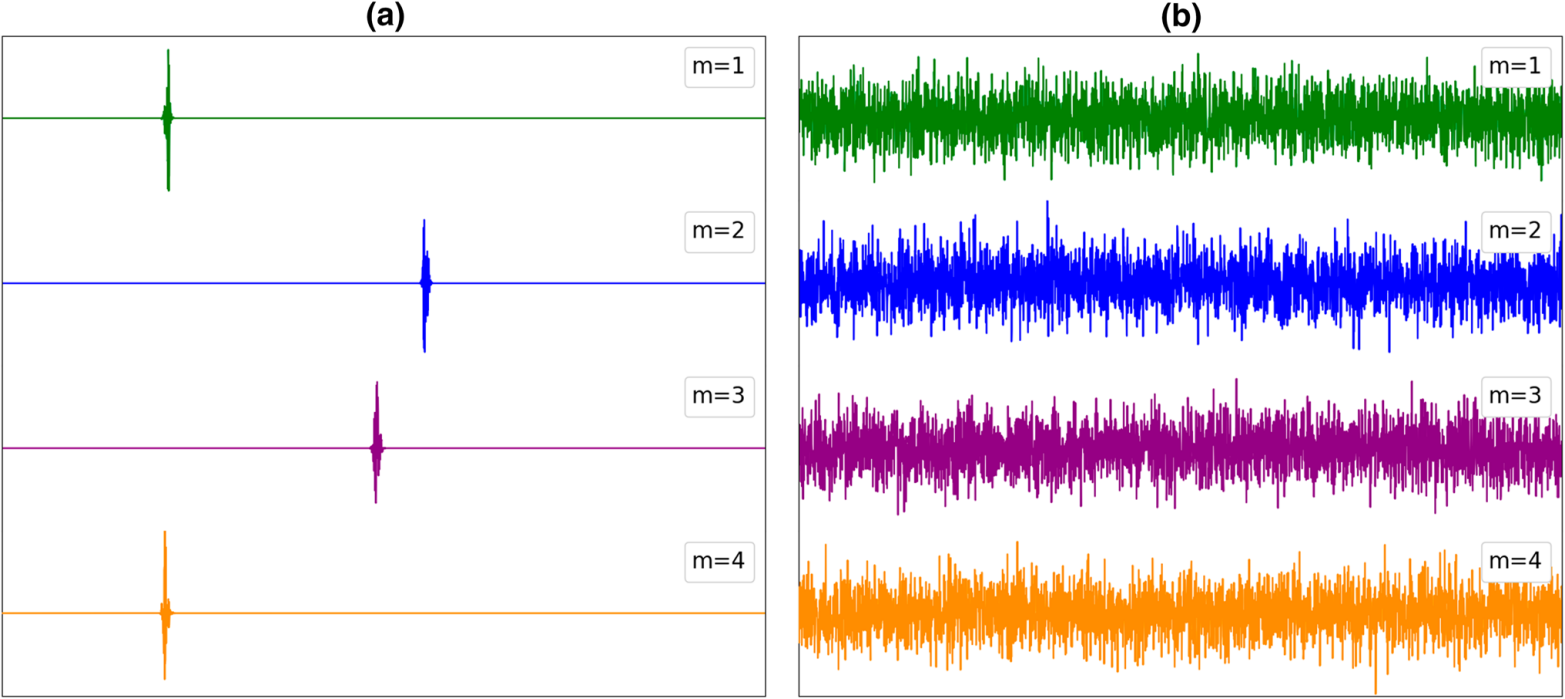
First four opened modes ${\widehat{u}}^{(m)}$ (corresponding to the first, second, third, and fourth largest eigenvalue) for **a** four first neighbors, and **b** N coupled neighbors (infinite interaction range). ${J}_{ij}\in \left[- 1, 1\right],$ 1D, *N* = 3000. The plot shows values of ${u}_{i}^{(m)}$ along the *x*-axis. The vertical axis is scaled in arbitrary units (i.e., $\sum_{i}{u}_{i}^{(m)2}=1)$

In Fig. 1 (${J}_{ij}\in [\mathrm{0,1}]$) and Fig. 2 (${J}_{ij}\in [-\mathrm{1,1}])$, we plot the first four modes (corresponding to the first, second, third, and fourth largest eigenvalue ${\Omega }_{m}$) that open on decreasing $\Omega$, where the number of interacting neighbors equals to (a) 4, and (b) *N*. One sees that for both “glass” and “spin-glass” matrices, these modes are localized for the case (a). Note that, the overlap between these clusters is in general negligible (see Figs. 1a, 2a). Therefore, in these cases, the modes are effectively decoupled and do not hinder each others growth if the relevant parameter (in this case $\Omega$) varies. On the contrary, if the range of interactions is large enough, all the modes are extended, see Figs. 1b, 2b. Consequently, in this case, the modes are strongly coupled.

**Fig. 3:**
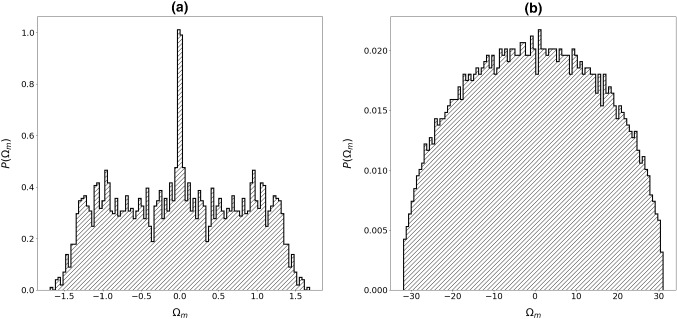
Distribution of eigenvalues for **a** first neighbors, **b** *N* coupled neighbors (infinite interaction range). ${J}_{ij}\in \left[0, 1\right],$ *N* = 3000

**Fig. 4:**
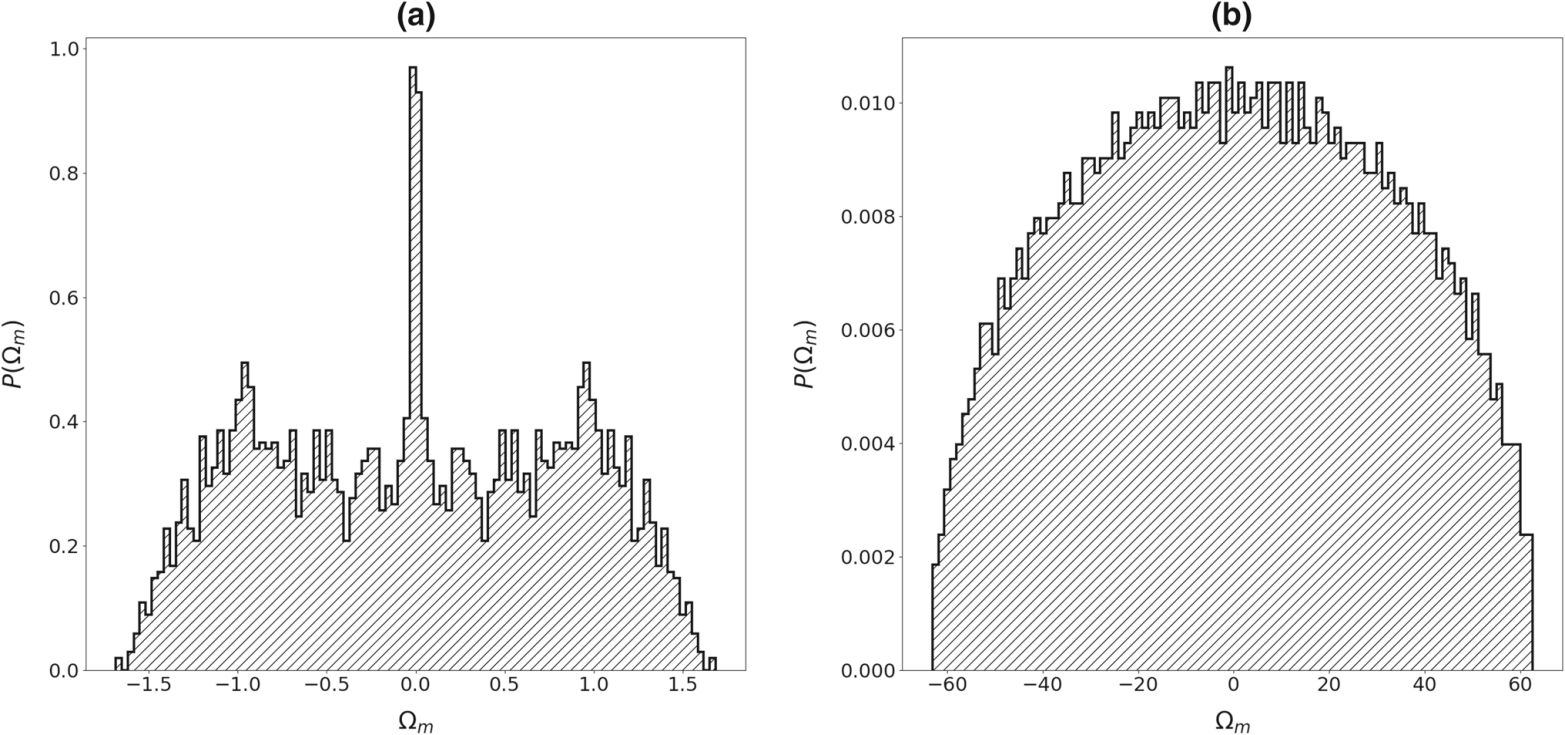
Distribution of eigenvalues for **a** first neighbors, **b** *N* coupled neighbors (infinite interaction range). ${J}_{ij}\in \left[- 1, 1\right],$ *N* = 3000

**Fig. 5:**
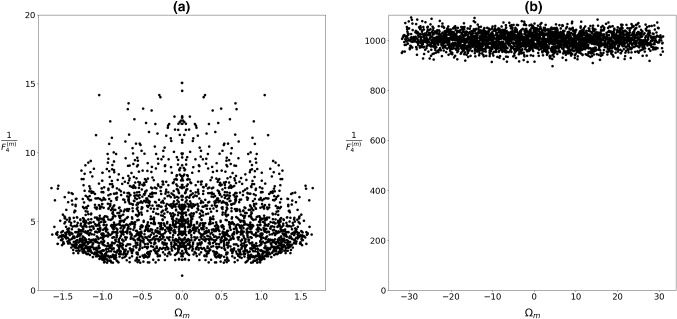
Values ${1/F}_{4}^{(m)}$ corresponding to eigenvalues ${\Omega }_{m}$ which roughly reflects number ${N}^{(m)}$ of *spins* within the *m*-th “cluster.” ${J}_{ij}\in \left[0, 1\right],$ *N* = 3000

**Fig. 6:**
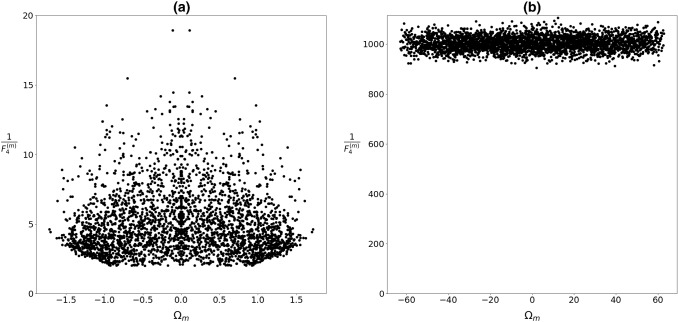
Values ${1/F}_{4}^{(m)}$ corresponding to eigenvalues ${\Omega }_{m}$ which roughly reflect number ${N}^{(m)}$ of *spins* within the *m*-th “cluster.” ${J}_{ij}\in \left[-\mathrm{1,1}\right],$ *N* = 3000

Note that, the general features of “glass” and “spin-glass” matrices are similar. This is well manifested in the distribution of eigenvalues $P({\Omega }_{m})$ (Figs. 3, 4) and values of ${1/F}_{4}^{(m)}$ (Figs. 5, 6) as a function of ${\Omega }_{m}.$ In the case of short-range interactions (first neighbors and 1D), both $P({\Omega }_{m})$ distributions exhibit a singularity at ${\Omega }_{m}=0.$ It fingerprints appearance of nonlocalized states. This anomaly was first observed by Weissmann [41] who studied electron states in the tight-bounding approximation with randomly distributed atoms within the crystal lattice, which illustrates the universality of the phenomenon. In fact, this phenomenon is related to the “famous” Anderson localization [42, 43]. The character of modes strongly changes on varying the number of neighbors. For a larger number of neighbors, the central anomaly in $P({\Omega }_{m})$ vanishes and nonlocalized states appear at finite values of ${\Omega }_{m}.$ Note that, the character of states could be well inferred from ${1/F}_{4}^{(m)}$. Figures 5b, 6b illustrate that for the infinite range of interactions, all the modes are nonlocalized. The corresponding distribution of eigenvalues is in this case well described by the Wigner distribution [22]

38 $$ P\left( {\left| {\Omega_{m} } \right| < \left| {\Omega_{1} } \right|} \right) = \frac{2}{{\pi \Omega_{1} }}\sqrt {\Omega_{1}^{2} - \Omega_{m}^{2} } . $$

## Discussion

We consider possible origins of domain-type configurations that are metastable or arrested on macroscopic time scales, exhibiting glass-like features, in systems that in “normal” conditions exhibit long-range orientational order in a temperature-driven symmetry-breaking phase transition. We present qualitatively different domain generating mechanisms in such systems, that might also appear mutually. These are (i) fast enough quenches, described by the universal Kibble-Zurek (KZ) mechanism [1], (ii) supercooling phase transformations, described by the Kibble mechanism [6], and (iii) Imry-Ma mechanism [15].

Note that, the KZ mechanism was originally derived for the second-order phase transition. However, it could be applied also to weakly first-order phase transition, e.g., isotropic-nematic LC phase transition [30]). The necessary condition is that the “freezing” condition is realized (i.e., one can introduce the Zurek time, see Eq. (4)). This requirement supplements the two conditions for the appearance of the Kibble mechanism: the existence of continuous symmetry-breaking phase transition and the finite speed of information propagation. The characteristic linear size of resulting protodomains, that are nucleated below the phase transition temperature is determined by the quench rate. Note that, after the domains “unfreeze,” they start to grow. In the presence of impurities, which are coupled to the relevant phase ordering field [4], an effective random-field could be stabilized if the coupling is strong enough [20]. Consequently, the domain pattern could persist in a metastable state.

Similarly, domain-type structures could appear in supercooled first-order phase transitions. In this case, we define the reduced temperature as $r=\frac{T-{T}^{*}}{{T}^{*}},$ where ${T}^{*}$ stands for the supercooling temperature. These systems should experience low enough perturbations so that supercooling is possible. In such cases, a system experiences local jumps into the ordered phase well below the equilibrium phase transition temperature ${T}_{c}$, where the energy barrier between the competing phases could be overcome via thermal fluctuations. Owing to the universal Kibble mechanism, domain pattern is expected. In this case, domains might remain arrested even in relatively pure samples, where impurities are weakly coupled with the relevant order parameter. Namely, at low enough temperature, the system might be stiff enough to enable unsurmountable energy barriers [44].

The potential stabilization of domains strongly depends on the symmetry of the order parameter [4]. Structures described by vector order parameters could exhibit point defects. On the contrary, the quadrupolar symmetry allows also line defects. Domain pattern’s growth is enabled by the annihilation of appropriate defects and antidefects. If line defects are present, this process is less probable. The resulting domains tend to grow with time [13, 14, 25], however, in the presence of impurities, their growth could be arrested. Impurities could strongly pin topological defects or even create additional TDs. This is well manifested in recent studies in LC phases perturbed by colloids [45–47], nanoparticles [48–52], or by surface imperfections [53]. The fundamental ingredient stabilizing and pinning topological defects seem to be curvature [54–63] and elastic terms supporting excitations exhibiting Gaussian curvature in a relevant physical field [64–71].

The resulting domain pattern might be presented as an ensemble of randomly interacting *effective spins*, where the latter represents an average orientation within a domain. Within such a complex system, the interaction among the *spins* is strongly influenced by topological defects that are formed at domain boundaries. Essential universal energy-related features of resulting configurations can be described by random matrices. The corresponding eigenvectors of matrices reveal patterns that such interaction intrinsically favors. Their character might have a strong impact on the resulting structural changes when relevant control parameters are changed (e.g., temperature). Note that, several studies reveal that the essential behavior of such systems is weakly dependent on detail interaction character [34–36]. For this reason, we use a relatively simple description, where the scaled interaction strength of mutually coupled spins either varies within the interval [− *J*, *J*] or [0, *J*], where *J* > 0 describes the maximal coupling strength favoring local parallel orientation of interacting units. These cases roughly mimic the behavior in systems exhibiting polar and quadrupole interactions, respectively. Of particular importance are eigenvectors corresponding to the largest matrix eigenvalue [36]. Namely, in general, they correspond to configurations that exhibit minimal energy penalty. The essential properties of these states are determined by the range of interactions within a system and spatial dimensionality, which both increase the number of mutually interacting system elements. In our model via mimic these effects just by increasing the number of interaction neighbors. The key features of such systems are as follows. In systems where few elements interact by random interactions display localized eigenvalues occur. On varying relevant parameters, one expects that different localized eigenmodes could be opened because the overlap between the competing modes is expected to be relatively low. Such systems are expected to show gradual non-critical evolution of order on varying relevant control parameters (e.g., temperature). On the other hand, if eigenmodes are nonlocalized, a critical-like behavior might be observed. Namely, the firstly opened mode is expected to hinder the opening of other competing modes, which promote different configurations.

## Conclusions

In the paper, we summarized common conditions that might stabilize domain-type structures in systems, which are under normal conditions expected to exhibit spatially homogeneous orientational order. In such systems, one expects several universal features which depend on the symmetry of order parameter and range of effective interactions within the system. We showed that domains are almost inevitable formed in continuous symmetry-breaking phase transitions if either quenched or strongly supercooled systems. In the former case, impurities are needed to pin topological defects and consequently stabilize domains. In the latter case, which appears only in first-order phase transitions, structures could be stabilized also if stiff enough order is formed. In either case, stabilization of domains is more effective if line defects could exist. The resulting general character of domain-type configurations could be inferred from behavior of random matrices, which mimic energetics of such structures entangled by essentially random network of topological defects. The related eigenvectors, corresponding to relatively large eigenvalues, are in general expected to have the strongest impact. If these states are localized, one expects gradual evolution of order on varying a relevant control parameter (*e.g.*, temperature). If the states are extended (nonlocalized), critical-like behavior might emerge. We plan to perform research to illustrate these effects also quantitatively by matching results of selected well-defined experimental systems and numerical studies using well-defined model which is designed to model experimentally measured quantities.

## Data Availability

Raw data are available by personal request.
